# Biocontrol Potential of *Bacillus velezensis* xd3 in Alleviating Root Rot of Garlic (*Allium sativum* L.)

**DOI:** 10.3390/jof12100721

**Published:** 2026-09-24

**Authors:** Yutao Zhu, Xidie Liu, Meizi Han, Xianyang Wang, Jiangnan Zhou, Ganlin Tang, Tao Zhu, Mei Zhao, Taotao Li, Chunli Liao, Peifang Ma, Donghai Chen, Lianzhe Wang

**Affiliations:** 1 Engineering Technology Research Center for Green Prevention and Control of Pests on Characteristic Crops in Pingdingshan, College of Life Science and Engineering, Henan University of Urban Construction, Pingdingshan 467036, China; 17516557933@163.com (X.L.); 17638213933@163.com (M.H.); 13839726250@163.com (X.W.); 17516598687@163.com (J.Z.); 15753972901@163.com (G.T.); 30110201@hncj.edu.cn (T.Z.); meizhao2021@163.com (M.Z.); ltt198906@163.com (T.L.); liao20130427@163.com (C.L.); 2 Pingdingshan Academy of Agricultural Sciences, Pingdingshan 467003, China; 15637530201@163.com; 3 Zhengzhou Academy of Agricultural and Forestry Science, Zhengzhou 450006, China; 362806722@163.com

**Keywords:** *Allium sativum* L., *Fusarium proliferatum*, root rot, *Bacillus velezensis*, biological control

## Abstract

Garlic root rot, mainly caused by *Fusarium proliferatum*, severely compromises yield and quality and causes substantial economic losses worldwide. Biocontrol using antagonistic bacteria has emerged as an efficient and sustainable strategy for managing this soil-borne disease. In this study, *Bacillus velezensis* xd3 was isolated and exhibited strong antagonistic activity against eight phytopathogenic fungi, with *F. proliferatum* as the representative pathogen. Scanning electron microscopy showed that treatment with the cell-free filtrate of *B. velezensis* xd3 induced prominent morphological abnormalities in *F. proliferatum* mycelia, including collapse, shrinkage, and distortion. Greenhouse pot assays further verified that *B. velezensis* xd3 significantly alleviated garlic root rot symptoms, achieving a biocontrol efficacy of 59.85%. Biochemical assays confirmed that *B. velezensis* xd3 is capable of secreting cellulase, amylase, protease, and siderophores. In addition, untargeted metabolomic profiling of the fermentation supernatant putatively indicated that *B. velezensis* xd3 may produce antifungal metabolites including lipopeptides (surfactins b and c), polyketides, and siderophores. Transcriptomic data revealed that *B. velezensis* xd3 exerts antifungal effects against *F. proliferatum* by modulating the expression of genes associated with membrane structure, transmembrane transport, and redox homeostasis. Collectively, these findings demonstrate that *B. velezensis* xd3 is a promising biocontrol candidate for the sustainable management of garlic root rot disease.

## 1. Introduction

Garlic (*Allium sativum* L.) is a perennial plant of the genus *Allium* in the Amaryllidaceae family, is widely cultivated across the globe and possesses considerable nutritional and medicinal value [1]. As an essential culinary condiment and functional food, garlic is rich in human-beneficial nutrients, including dietary fiber, vitamin C, allicin, polysaccharides and minerals [2]. Accumulating phenolic compounds, flavonoids, and organosulfur endow garlic with diverse biological properties, such as anticancer, antiviral, antibacterial, cardioprotective, and nephroprotective activities [3,4]. In 2024, global garlic production reached 29.69 million tons, with China (21.65 million tons) accounting for 72.92% of the total global output, indicating that garlic holds significant economic and market value [5].

In recent years, the incidence of garlic root rot has been continuously rising across China, resulting in approximately 20–30% yield losses, with severe epidemic areas suffering yield reductions of over 50% [6]. The primary pathogenic fungi causing root rot are *Fusarium* species, such as *Fusarium oxysporum*, *Fusarium solani*, and *Fusarium proliferatum*, which can infect plant roots, stem bases, and bulb tissues [7,8]. Soil and garlic seed bulbs serve as the major transmission vectors of *Fusarium* pathogens, which can infect garlic throughout the entire growth period [7]. After successful colonization, *Fusarium* species produces toxins such as fumonisins, moniliformin, and fusaric acid, which are toxic to both humans and animals [9]. Nevertheless, the long-term overapplication of chemical agents inevitably causes environmental contamination, induces the emergence of pathogen resistance, and poses potential risks to human health [10]. The utilization of beneficial microorganisms for biological control offers advantages such as environmental friendliness and ecological safety, making it a highly promising strategy for plant disease management in modern green agriculture [8,11].

*Bacillus* is a ubiquitously distributed bacterial genus with well-documented potential for the biocontrol of plant root rot diseases. For example, *Bacillus subtilis* CG-6 strengthens the activities of antioxidant and defense-related enzymes in alfalfa, thereby effectively suppressing root rot caused by *F. oxysporum* [12]. *Bacillus amyloliquefaciens* FS6 can significantly inhibit the mycelial growth of *Sclerotinia* spp., presenting a field control efficacy of 53.00–73.51% against ginseng root rot [13]. The strain *Bacillus mojavensis* dxk33 alleviates root rot damage in *Cunninghamia lanceolata* by inhibiting the proliferation of *F. solani* and reshaping beneficial rhizosphere bacterial communities [14]. Notably, *Bacillus velezensis* Yao exhibits a dual biocontrol mechanism: it produces lipopeptide compounds to suppress the mycelial growth of *F. solani*, and simultaneously activates plant pattern-triggered immunity (PTI) and effector-triggered immunity (ETI) signaling pathways, ultimately improving pepper resistance to root rot [11]. Beyond the above cases, *Bacillus* antagonistic strains also possess excellent biocontrol performance against root rot in a variety of economic and crop plants, including tomato [15], soybean [16], *Coptis chinensis* [17], kiwifruit [18], *Lonicera macranthoides* [10], and potato [19].

Currently, biocontrol bacteria against garlic root rot, such as *B. amyloliquefaciens* [20] and *B. siamensis* [21], have been successively reported. However, there is an urgent need to explore and isolate more biocontrol bacteria to combat garlic root rot. This study aims to explore the biocontrol efficacy of *B. velezensis* xd3 against root rot caused by *F.proliferatum* in garlic and to elucidate its antifungal mechanism. *B. velezensis* xd3 exhibits significant inhibitory effects on eight plant pathogenic fungi, including *F. proliferatum. B. velezensis* xd3 treatment induced severe morphological abnormalities in the mycelia of *F. proliferatum*. Under greenhouse conditions, strain xd3 markedly alleviated garlic root rot symptoms. Metabolomic profiling tentatively suggests that *B. velezensis* xd3 may secrete antimicrobial substances, including lipopeptides, polyketides, and siderophores. Finally, transcriptomic analysis further deciphered the molecular mechanisms underlying the inhibitory effect of *B. velezensis* xd3 on *F. proliferatum*. Collectively, these findings provide a solid theoretical basis for the future development and application of *B. velezensis* xd3 as a promising biocontrol agent against garlic root rot.

## 2. Materials and Methods

### 2.1. The Pathogenic Fungus

The highly pathogenic garlic root rot pathogen *F. proliferatum* was preserved in the laboratory. The fungus was subcultured on PDA medium and incubated at 28 ± 2 °C for 5 days.

### 2.2. Isolation and Screening of Antagonistic Bacteria Against F. proliferatum

Bacterial strains were isolated from the rhizosphere soil of cultivated morel mushrooms (Shangqiu City, Henan Province, China) using the soil-dilution plating method. Briefly, 1 g of fresh soil sample was suspended in 9 mL of sterile distilled water, and the mixture was shaken continuously at 200 rpm for 30 min to obtain a homogeneous soil suspension. The suspension was then serially diluted (up to 10^−5^) and spread onto Luria–Bertani (LB) solid medium, followed by incubation at 28 ± 2 °C for 24 h. Bacterial colonies grown on the plates were picked and purified based on their colony morphological features. Isolates were maintained on LB slant medium at 4 °C for subsequent experiments.

The antagonistic activity of biocontrol strains against *F. proliferatum* was determined using the potato dextrose agar (PDA) plate confrontation assay. An 8 mm mycelial plug of *F. proliferatum* was inoculated in the center of the medium, and filter paper discs containing 5 μL of biocontrol strain suspension (OD_600_ = 0.8) were placed at 2 cm intervals from the PDA center in four directions (up, down, left, right). PDA plates without biocontrol strains served as controls, and all plates were incubated at 28 °C for 7 days. The inhibition rate was calculated using the following formula [12]: inhibition rate = (C − T)/(C − C_0_) × 100%, where C is the radial growth of fungus in the control, T is the same in dual culture, and C_0_ is the diameter of the test fungus mycelial plug (8 mm). Biological control strains with an inhibition rate exceeding 50% were used for further experiments.

### 2.3. Determination of xd3 Antifungal Spectrum

Strain xd3, which showed the strongest inhibitory activity against *F. proliferatum*, was selected to evaluate its broad-spectrum antimicrobial property. Seven phytopathogenic fungi preserved in our laboratory were used for the assay, including *Fusarium pseudograminearum* (wheat crown rot), *Fusarium oxysporum* (cotton fusarium wilt), *Fusarium equiseti* (soybean root rot), *Rhizoctonia solani* (rice sheath blight), *Alternaria alternata* (tobacco brown spot), *Sclerotium rolfsii* (peanut southern blight), and *Botrytis squamosa* (leek gray mold). The inhibition rates were determined following the procedure described in Section 2.2.

### 2.4. Identification of the Biocontrol Bacterium xd3

Total genomic DNA was extracted from bacterial cells using the M5 HiPer Bacteria Genomic DNA Kit (Mei5 Biotechnology Co., Ltd., Beijing, China). The 16S rRNA gene was amplified using primers 27F (5′-AGAGTTTGATCCTGGCTCAG-3′) and 1492R (5′-TACGGCTACCTTGTACGACTT-3′), while the *gyrA* gene was amplified with primers *gyrA*-F (5′-ACAGTCAGGAAATGCGTACGTCCTT-3′) and *gyrA*-R (5′-CAAGGTAATGCTCCAGGCATTGCT-3′). The PCR mixture was prepared in a total volume of 20 μL, containing 1 μL DNA template, 0.5 μL of each forward and reverse primer, 10 μL PCR mix, and ddH_2_O. The PCR amplification program was set as follows: initial denaturation at 95 °C for 3 min; 32 cycles consisting of denaturation at 94 °C for 25 s, annealing at 55 °C for 25 s, and extension at 72 °C for 15 s, followed by a final extension step at 72 °C for 5 min. The amplification products were purified by 1.2% agarose gel electrophoresis and sequenced by General Biosystems Co., Ltd. (Chuzhou, China). Homology analysis was performed using the GenBank database through the BLAST program in National Center for Biotechnology Information (NCBI). Phylogenetic and molecular evolutionary analyses were conducted using the neighbor-joining method with MEGA 6.0 [22].

### 2.5. Analysis of Biocontrol Characteristics of xd3 Strain

Siderophore production by the xd3 strain was qualitatively detected according to the method reported by Schwyn and Neilands [23]. Briefly, 5 μL of the activated xd3 bacterial suspension (OD_600_ = 0.8) was inoculated at the center of a chrome azurol sulphonate (CAS) solid agar plate and cultured at 28 °C in darkness for 3 days. The formation of orange halos around bacterial colonies was then observed and recorded. The extracellular enzyme activities (protease, amylase, and cellulase) of the xd3 strain were determined qualitatively following the protocol described by PalaniArul et al. [24]. Similarly, 5 μL of the activated xd3 bacterial suspension (OD_600_ = 0.8) was spot-inoculated on corresponding selective media and incubated at 28 °C under dark conditions for 3 days. The formation of a transparent zone (lysis zone) around the colony indicates that the xd3 strain possesses the characteristic of secreting this enzyme. The IAA production capacity of strain xd3 was determined via the Salkowski colorimetric method [25]. The strain xd3 was cultured in LB medium amended with 1 g/L tryptophan at 28 °C for 48 h. After incubation, the bacterial culture was centrifuged to collect the supernatant. Subsequently, 0.2 mL of the supernatant was mixed with an equal volume of Salkowski reagent, and the mixture was incubated in darkness for 30 min. The appearance of a pink color in the reaction solution indicated IAA production. Uninoculated LB medium was set as the negative control, and a 50 mg/L IAA aqueous solution was used as the positive control in this assay.

### 2.6. Effects of xd3 Strain on Mycelia Morphology of F. proliferatum

Strain xd3 was cultured in LB medium with shaking incubation at 28 °C for 48 h. The resulting bacterial suspension was centrifuged at 12,000× *g* rpm for 10 min at room temperature, and the supernatant was recovered before being sterile-filtered through a 0.22 μm microporous membrane. The obtained cell-free filtrate was mixed with PDA medium at a volume ratio of 20% (*v*/*v*). *F. proliferatum* mycelial plugs were placed in the centre of the amended PDA plates, followed by incubation at 25 °C for 7 d. The inhibitory effect of the sterile filtrate on the vegetative growth of *F. proliferatum* was subsequently assessed after incubation.

For the sample preparation for scanning electron microscopy (SEM), fungal hyphae were collected after treatment with the cell-free sterile filtrate of strain xd3 and rinsed three times with sterile phosphate-buffered saline (PBS, pH 7.2). The hyphal specimens were fixed overnight at 4 °C in 2.5% (*v*/*v*) glutaraldehyde solution. After primary fixation, samples were washed three times using PBS, followed by post-fixation with 1% osmium tetroxide for 2 h at room temperature. The hyphae were then subjected to serial ethanol dehydration in a graded ethanol series (30%, 50%, 70%, 80%, 90%, and 100%), with each concentration maintained for 15 min. Upon completion of dehydration, the samples were transferred to tertiary butyl alcohol for replacement and dried using a vacuum freeze-dryer. The dried specimens were affixed onto conductive adhesive tape and sputter-coated with a thin gold layer. Ultimately, the micromorphology of fungal hyphae was observed and photographed under a scanning electron microscope at an accelerating voltage of 15 kV. All treatments were conducted with three independent biological replicates to guarantee the reproducibility of morphological observations.

### 2.7. UHPLC-MS Analysis of Antifungal Metabolites

The antimicrobial metabolites produced by strain xd3 were characterized via ultra-high-performance liquid chromatography coupled with high-resolution mass spectrometry (UHPLC-HRMS) (General Biol (Anhui) Co., Ltd., No.69 Qifusi West Road, Chuzhou Economic-Technological Development Zone, Chuzhou 239000, Anhui, China). Strain xd3 was cultivated in liquid fermentation medium at 28 °C with orbital shaking at 180 rpm for 48 h, and three independent biological replicates were included. Uninoculated fermentation medium was set as the medium blank control. An exact volume of 2 mL of sterile fermentation broth was blended with pre-cooled methanol/acetonitrile/water (2:2:1, *v*/*v*/*v*), followed by vortex oscillation for metabolite extraction. The mixture was transferred to a fresh centrifuge tube and centrifuged at 14,000 *g* for 20 min, after which the supernatant was harvested. The supernatant was concentrated to complete dryness in a vacuum concentrator. Before UHPLC-HRMS detection, the dried residues were reconstituted with 100 μL acetonitrile–water mixture (1:1, *v*/*v*). After centrifugation at 14,000 *g* and 4 °C for 15 min, the resultant supernatant was transferred for instrumental injection and analysis.

Analyses were conducted on a Vanquish UHPLC system (Thermo Fisher Scientific) coupled with an Orbitrap Exploris™ 480 mass spectrometer at Shanghai Applied Protein Technology Co., Ltd., No. 58 Yuanmei Road, Minhang District, Shanghai 201109, China. Separation was achieved via hydrophilic interaction liquid chromatography (HILIC) using an ACQUITY UPLC BEH Amide column (2.1 mm × 100 mm, particle size 1.7 μm; Waters, Ireland). Analyses were carried out under both electrospray ionization positive (ESI^+^) and negative (ESI^−^) modes. The mobile phase consisted of mobile phase A: aqueous solution containing 25 mM ammonium acetate and 25 mM ammonium hydroxide, and mobile phase B: pure acetonitrile. The elution gradient program was set as follows: maintained at 95% mobile phase B for 0.5 min; linearly decreased to 65% B within 6.5 min; further lowered to 40% B over 1 min and held for 1 min; ramped back up to 95% B in 0.1 min, followed by an equilibration hold for 2.9 min.

ESI source parameters (Thermo) were configured as follows: sheath gas flow rate = 50 Arb, auxiliary gas flow rate = 2 Arb, capillary temperature = 350 °C, spray voltage = +3500 V (positive mode)/−2800 V (negative mode). For full-scan MS acquisition, the scanning m/z range was defined as 70–1200 Da, the resolution was set to 60,000, and the accumulation time was 100 ms. For automated MS/MS acquisition, the *m*/*z* range, resolution, and accumulation time remained consistent with those used for full-scan MS, with a precursor exclusion time of 4 s. Metabolites were identified by matching accurate *m*/*z* values (mass tolerance < 10 ppm) and MS/MS spectra against the authentic standard spectral database from General Biology (Anhui) Co., Ltd. 

### 2.8. Greenhouse Pot Experiments

Pot experiments were carried out using garlic cultivar Yusuan No. 1. Garlic seedlings were grown in plastic pots (8 cm in diameter and 7 cm in height) filled with a mixed substrate of nutrient soil and vermiculite at a volume ratio of 1:1. For pathogen inoculation, the roots of 10-day-old garlic seedlings were inoculated with *F. proliferatum* conidial suspension (1 × 10^7^ conidia/mL). Immediately after pathogen root irrigation, the garlic seedlings were subjected to root irrigation with 10 mL of xd3 bacterial suspension (OD_600_ = 0.8), and seedlings treated with LB medium served as the blank control group. Each treatment contained three independent biological replicates, with at least 15 garlic seedlings per replicate. All garlic plants were cultured in a greenhouse under controlled conditions at 25 °C with a 16 h light photoperiod. Disease symptoms were observed after 21 days, and the severity of garlic root rot at the seedling stage was classified as follows [26]:

Grade 0: No disease symptoms present. Grade 1: Water-soaked lesions cover less than 15% of root area. Grade 3: Water-soaked appearance appears at stem base, with water-soaked lesions covering 15–50% of root area. Grade 5: Obvious water-soaked and softened stem base, with water-soaked lesions covering over 50% of root area. Grade 7: Severe rotting at stem base, with water-soaked lesions covering the entire root system.

The plant disease index (DI) was calculated using the formula: DI = [Σ (n × number of seedlings at level n)/(7 × number of total seedlings)] × 100, where n denotes the disease levels from 0 to 7. Relative control efficacy = (Disease index of control—Disease index of treatment)/Disease index of control × 100%.

### 2.9. Transcriptomics of xd3 Strain Against F. proliferatum

Transcriptome sequencing combined with bioinformatic analysis was performed to investigate the response mechanism of *F. proliferatum* against strain xd3. Briefly, a mycelial plug of *F. proliferatum* was inoculated at the centre of PDA medium, and filter-paper discs loaded with antagonistic strain xd3 were placed around the fungal plug. PDA plates without xd3 treatment were established as the control group. After incubation at 25 °C for 7 days, fungal mycelia were harvested and immediately frozen in liquid nitrogen. Three independent biological replicates were set for each treatment.

A total of 1 μg of total RNA isolated from mycelial samples was utilized for RNA-seq library construction. High-throughput paired-end sequencing (2 × 150 bp) was performed on the Illumina NovaSeq platform (Illumina, San Diego, CA, USA). Raw FASTQ sequencing reads were preprocessed with fastp v0.20.1 (https://github.com/OpenGene/fastp) (accessed on 22 July 2026) [27] to eliminate technical adapter sequences and low-quality bases. The resulting clean reads were mapped to the *F. proliferatum* reference genome (ASM2262713v1) [28] using STAR v2.7.7a (https://github.com/alexdobin/STAR) (accessed on 22 July 2026) [29]. Mapped reads were counted for each gene with FeatureCount v2.0.0 (https://github.com/topics/featurecounts) (accessed on 24 July 2026). StringTie was employed to assemble transcripts and convert processed sequencing data into a gene transcript expression matrix for the quantification of gene expression levels [30]. The raw read-count matrix was imported into EdgeR for differentially expressed gene (DEG) analysis (https://bioconductor.org/packages/release/bioc/html/edgeR.html) (accessed on 24 July 2026) with the threshold of FDR < 0.05 and |log_2_(fold change)| ≥ 1 [31]. Functional enrichment analyses of DEGs were conducted against Gene Ontology (GO) terms and Kyoto Encyclopedia of Genes and Genomes (KEGG) pathways to dissect their biological implications [32].

### 2.10. Validation of Gene Expression in F. proliferatum by Quantitative Real-Time PCR

To verify the reliability of transcriptomic data, twelve genes of *F. proliferatum* were randomly selected for qRT-PCR analysis. Total RNA was extracted from mycelia using an RNA extraction kit, and cDNA was subsequently synthesized via a reverse transcription kit (Mei5 Biotechnology, Co., Ltd., Beijing, China). qRT-PCR reactions were carried out on a QuantStudio 6 Pro real-time PCR system (Applied Biosystems, Foster City, CA, USA) employing 2 × M5 HiPer Realtime PCR Super mix with Low ROX (SYBR Green, containing anti-Taq antibody) (Mei5 Biotechnology Co., Ltd., Beijing, China). EF1α (MW161161.1) was selected as the reference gene for cDNA normalization to quantify gene expression levels [33]. Relative gene expression abundances were calculated via the 2^−ΔΔCt^ method [34]. All qRT-PCR reactions were carried out with three independent biological replicates. Primer sequences used in this study are summarized in Supplementary Table S1.

### 2.11. Data Analysis

Data were obtained from three independent replicates per treatment and are presented as mean ± standard deviation. Significant differences between groups were compared with a confidence level of *p* < 0.05 by Duncan’s multiple range test using statistical software IBM SPSS Statistics 20.

## 3. Results

### 3.1. Isolation and Identification of the Antifungal Bacterial Strain xd3

In this study, a total of 120 bacterial strains were isolated from the rhizosphere soil of cultivated morel mushrooms. Among these isolates, strain xd3 exhibited the most potent inhibitory activity against *F. proliferatum*, with an inhibition rate exceeding 70%. On LB medium, the xd3 colonies appeared milky white and opaque, with clear but irregular edges. The colony surface was dry and wrinkled, emitting a distinctive odor (Figure S1A). Gram staining revealed that xd3 cells exhibited a bluish-purple color, identified as a Gram-positive rod-shaped strain (Figure S1B). The 16S rRNA (accession number QB023163) and *gyrA* gene sequences of strain xd3 are provided in Table S2. Phylogenetic analysis based on the 16S rRNA sequence revealed that xd3 shares high homology with *Bacillus velezensis* XYDJ2 and clusters within this species in the phylogenetic tree (Figure 1A). Furthermore, the phylogenetic tree constructed based on the *gyrA* gene revealed that strain xd3 clustered together with *B. velezensis* within a single clade (Figure 1B). Combined results from colonial morphology and molecular identification confirm that strain xd3 belongs to *B. velezensis*.

### 3.2. Antimicrobial Spectrum of B. velezensis xd3

*B. velezensis* strain xd3 exhibited prominent antifungal performance and significantly suppressed the mycelial growth of eight economically important phytopathogenic fungi (Figure 2). The inhibition rate of *B. velezensis* strain xd3 against all eight fungi exceeded 69% (Table 1). These results indicate that *B. velezensis* strain xd3 exhibits broad-spectrum antifungal activity and has potential for biocontrol of diverse plant fungal diseases.

### 3.3. Effect of B. velezensis xd3 on the Morphology of F. proliferatum

To determine the origin of antifungal metabolites, we assessed the inhibitory activity of cell-free fermentation broth from *B. velezensis* strain xd3 against *F. proliferatum*. Compared with the control group, the mycelial growth of *F. proliferatum* was markedly retarded on medium amended with 20% (*v*/*v*) cell-free fermentation broth (Figure 3A,B). In addition, SEM revealed obvious morphological changes in *F. proliferatum* hyphae following treatment with the cell-free fermentation broth of strain xd3. Hyphae from the control samples displayed normal growth with intact and well-organized mycelial architecture (Figure 3C). In contrast, hyphae exposed to the cell-free fermentation broth of *B. velezensis* strain xd3 exhibited severe distortion, shrinkage, atrophy and fragmentation (Figure 3D).

### 3.4. Efficacy of B. velezensis xd3 Against Garlic Root Rot Disease in Greenhouse

In greenhouse experiments, *B. velezensis* xd3 significantly reduced the severity of garlic root rot at 21 days post-inoculation with *F. proliferatum* (Figure 4). Garlic plants inoculated with *F. proliferatum* plus LB medium (CK) displayed prominent stunting, leaf yellowing, and obvious water-soaked symptoms on bulbous roots (Figure 4A,B). In contrast, garlic plants co-inoculated with *F. proliferatum* and strain *B. velezensis* xd3 grew well; only partial leaf desiccation was observed in a subset of plants (Figure 4C,D). The disease index reached 65.88% in the CK group, whereas it decreased to 26.45% in the *B. velezensis* xd3-treated group. *B. velezensis* xd3 achieved a biocontrol efficacy of 59.85% against garlic root rot under pot conditions (Table 2).

### 3.5. Antimicrobial and Plant Growth-promotion Traits of B. velezensis xd3

The antimicrobial and plant growth-promotion traits of *B. velezensis* xd3 were investigated via in vitro assays. Distinct transparent zones were detected around xd3 colonies, indicating that this strain can produce cellulase, amylase, and protease (Figure 5A–C). Siderophore production assays yielded distinct yellow transparent halos surrounding xd3 colonies, verifying its ability to secrete siderophores (Figure 5D). Furthermore, qualitative IAA production assays confirmed that *B. velezensis* strain xd3 is capable of synthesizing IAA (Figure 5E). Collectively, these results demonstrate that *B. velezensis* strain xd3 possesses excellent potential for biocontrol and plant growth promotion applications.

### 3.6. Analysis of Antifungal Metabolites in Sterile Fermentation Broth of Strain xd3

UHPLC-MS was used to analyze bioactive compounds from *B. velezensis* xd3. A total of 1737 metabolites were putatively annotated in the sterile fermentation broth of *B. velezensis* xd3. In terms of compositional proportion, miscellaneous metabolites accounted for 43.29%, amino acids and peptides 25.73%, unknown metabolites 12.15%, and carbohydrates 4.15% (Table S3 and Figure S2). Previous studies have demonstrated that *Bacillus* species typically secrete lipopeptides, polyketides, and siderophores to directly or indirectly suppress mycelial growth in phytopathogenic fungi [35,36,37]. The lipopeptides surfactin b and surfactin c yielded distinct chromatographic peaks at *m*/*z* 1022.67 (retention time: 272.62 s) and *m*/*z* 1036.69 (retention time: 270.47 s), respectively (Figure 6A,B), and were tentatively assigned on the basis of mass-spectral library matching. Moreover, the polyketides epothilone B and myriocin were tentatively annotated at *m*/*z* 490.26 (retention time: 230.47 s) and *m*/*z* 400.27 (retention time: 287.00 s), correspondingly (Figure 6C,D). Mass spectral peaks at *m*/*z* 654.28 (retention time: 417.33 s), 587.35 (retention time: 755.84 s), and 601.34 (retention time: 757.33 s) were tentatively assigned to ferrioxamine, desferrioxamine D2, and nocardamine, respectively; all these compounds are classified as siderophores (Figure S3). The full list of putatively annotated compounds together with their relative abundances is shown in Table 3.

### 3.7. Transcriptome Profiling Effects of B. velezensis xd3 on F. proliferatum

To understand the molecular mechanism of xd3 antagonizing *F. proliferatum*, we analyzed the transcriptome of *F. proliferatum* with or without *B. velezensis* xd3 treatment. The results showed that 1157 genes were upregulated and 794 genes were downregulated in *F. proliferatum* exposed to *B. velezensis* xd3 (Figure S4). According to GO analysis, these DEGs were related to biological processes (BP), cellular components (CC), and molecular functions (MF) (Figure S5). In the BP category, upregulated differentially expressed genes were primarily enriched in RNA processing (GO:0006396), ncRNA metabolic process (GO:0034660), and ribonucleoprotein complex biogenesis (GO:0022613). By contrast, downregulated differentially expressed genes were mainly enriched in transmembrane transport (GO:0055085), ion transport (GO:0006811), cellular response to chemical stimulus (GO:0070887), and oxidation–reduction process (GO:0055114). For the CC category, the upregulated differentially expressed genes were predominantly concentrated in ribonucleoprotein complex (GO:1990904), nucleolus (GO:0005730), and preribosome (GO:0030684), while the downregulated genes were enriched in endoplasmic reticulum (GO:0005783), plasma membrane (GO:0005886), and vacuole (GO:0005773). In the molecular function (MF) category, upregulated DEGs were mainly annotated to RNA binding (GO:0003723), RNA-targeted catalytic activity (GO:0140098), pyrophosphatase activity (GO:0016462), and acid anhydride hydrolase activity (GO:0016817). Meanwhile, downregulated genes were predominantly enriched in transporter activity (GO:0005215), transmembrane transporter activity (GO:0022857), oxidoreductase activity (GO:0016491), and inorganic molecular entity transmembrane transporter activity (GO:0015318) (Figure 7A,B). Overall, GO enrichment analysis suggested that treatment with *B. velezensis* xd3 may trigger transcriptional alterations in genes associated with RNA processing and energy metabolism, as well as pathways related to membrane structure, substance transport and exchange, and antioxidant defense systems in *F. proliferatum*.

KEGG enrichment analysis showed that the DEGs were mainly assigned to ribosome biogenesis (ko03009), transfer RNA biogenesis (ko03016), spliceosome (ko03041), translation factors (ko03012), ribosome biogenesis in eukaryotes (ko03008), and amino acid related enzymes (ko01007) (Figure S6). Among the pathways involving upregulated genes, the pathways enriched with a larger number of DEGs include ribosome biogenesis (ko03009), transfer RNA biogenesis (ko03016), chromosome and associated proteins (ko03036), spliceosome (ko03041), transcription machinery (ko03021), and translation factors (ko03012) (Figure 8A). In contrast, downregulated genes were enriched in transporters (ko02000), enzymes with EC numbers (ko99980), membrane trafficking (ko04131), exosome (ko04147), and mitochondrial biogenesis (ko03029). Furthermore, genes enriched in metabolism of xenobiotics by cytochrome P450, glutathione metabolism, chaperones and folding catalysts, MAPK signaling pathway, and mineral absorption were also significantly downregulated (Figure 8B). These findings suggest that treatment with *B. velezensis* xd3 may trigger transcriptional changes in genes associated with antioxidant defense, stress perception, and signal transduction in *F. proliferatum*.

### 3.8. qRT-PCR Validation of Randomly Selected Genes in F. proliferatum

To validate the RNA-Seq gene expression results, qRT-PCR was used to detect the expression of 12 randomly selected genes in *F. proliferatum*. All gene IDs and their corresponding encoded proteins are listed in Supplementary Table S4. Among them, three genes related to redox regulation (LB504_004467, LB504_006262, and LB504_006530) exhibited varying degrees of expression downregulation (Figure 9A–C). Two genes involved in transmembrane ion transport (LB504_001718 and LB504_011493) were also downregulated (Figure 9D,E). Furthermore, genes associated with the transport of siderophores (LB504_005354), amino acids (LB504_008673), and pyridoxine (LB504_012084) also showed reduced expression (Figure 9F–H). In contrast, genes participating in ribosomal protein assembly (LB504_000819 and LB504_006924), RNA modification (LB504_000868), and recognition and clearance of misfolded proteins (LB504_002411) were upregulated to varying degrees (Figure 9I–L). Comparison of qRT-PCR and transcriptome data revealed that, despite slight differences in gene expression magnitudes, the overall expression trends of up- and down-regulated genes were consistent, verifying the reliability of the transcriptome sequencing results in the present study.

## 4. Discussion

Garlic root rot caused by *Fusarium* spp. is one of the most destructive diseases of garlic, severely restricting the development of the garlic industry [7]. Although chemical pesticides exhibit remarkable efficacy for plant disease management, their excessive application may trigger the emergence of pathogen-resistant strains and cause environmental contamination, which highlights an urgent demand for alternative disease-control strategies [38,39]. In recent years, utilizing microorganisms for biological control has become an efficient, safe, and environmentally friendly strategy for managing various plant diseases [40]. At present, *B. amyloliquefaciens* [20], *B. siamensis* [21], *B. subtilis* [41] and *Streptomyces albidoflavus* [26] have been reported to possess biocontrol efficacy against garlic root rot. In the present study, *B. velezensis* xd3 displayed prominent antagonistic activity against eight phytopathogenic fungi, including *F. proliferatum* (Figure 2). Furthermore, pot experiments showed that garlic plants treated with xd3 exhibited significantly reduced leaf yellowing and bulb rot (Figure 4). These results suggest that *B. velezensis* xd3 can serve as a novel candidate microorganism for the biocontrol of garlic root rot.

Plate confrontation assays showed that strain xd3 markedly inhibited the colony growth of *F. proliferatum* (Figure 2A). Scanning electron microscopy (SEM) further revealed severe distortion, fracture, and shrinkage of *F. proliferatum* hyphae upon exposure to strain xd3. Collectively, these observations indicate that strain xd3 may secrete antifungal metabolites that disrupt normal developmental processes of this phytopathogen (Figure 3). It is well known that biocontrol bacteria of the genus *Bacillus* commonly produce various antimicrobial including antibiotics, lipopeptides, polyketides, siderophores and extracellular lytic enzymes, which inhibit mycelial growth or spore germination of plant-pathogenic fungi [35,36,42]. Chitinase, cellulase, amylase, and protease are important lytic enzymes that can degrade cell walls, thereby disrupting pathogen growth and spore germination [43,44]. Here, we found that strain xd3 secretes cellulase, amylase, and protease (Figure 5). Consistent with several other *Bacillus* species, these hydrolytic enzymes may constitute one set of active components contributing to its antagonistic activity against *F. proliferatum*. Lipopeptides such as fengycins, surfactins, and iturins are known to disrupt fungal membranes [45]. Previous studies have also confirmed that surfactin produced by *Bacillus* inhibits mycelial growth of *Botrytis cinerea* [46], *Magnaporthe oryzae* [47], *Fusarium oxysporum* [48], *Calonectria ilicicola* [49], and *Fusarium graminearum* [50]. In the present study, metabolomic profiling suggests that *B. velezensis* xd3 may produce putative surfactin (Figure 6), which could act as one of the antifungal compounds against *F. proliferatum*. Furthermore, the metabolomic data indicate that *B. velezensis* xd3 may also yield putative polyketides and siderophores, both of which have been reported to interfere with normal fungal growth [35,36]. Previous studies have demonstrated that *B. velezensis* Yao can secrete fengycin to efficiently inhibit *F. solani* [11]. The strain *B. velezensis* ARRI17 suppresses the growth of *Ilyonectria mors-panacis* via iturin [51]. Furthermore, *B. velezensis* NS13 [10] and GFB08 [52] interfere with the development of *Fusarium* spp. and *Colletotrichum* spp. through surfactin and fengycin, respectively. In the present study, putative surfactin, polyketides, and siderophores were tentatively annotated via mass-spectral library matching in the fermentation broth of *B. velezensis* xd3. These findings suggest that the antimicrobial metabolites produced by *B. velezensis* strains from different sources may vary considerably. As reported by Ibrahim et al., the potent antifungal activity of *B. velezensis* arises from the combined effects of siderophores, hydrolytic enzymes, and lipopeptides [53]. Collectively, *B. velezensis* xd3 produces hydrolases (verified by biochemical assays) as well as tentatively annotated lipopeptides, polyketides, and siderophores, which may jointly contribute to its favorable broad-spectrum antifungal activity. Nevertheless, further investigations are needed to determine which metabolite contributes the major antifungal effect.

To explore the molecular mechanism underlying the inhibitory effect of xd3 on *F. proliferatum*, we performed transcriptome sequencing on xd3-treated *F. proliferatum*, followed by analysis and validation of the associated DEGs. Transcriptomic data revealed that xd3 treatment substantially reshaped the global transcriptional profile of *F. proliferatum*, with 1157 upregulated genes and 794 downregulated genes. Specifically, the differentially expressed genes (DEGs) were associated with membranes, membrane transport, and redox homeostasis (Figure 7). These transcriptional changes raise the hypothesis that perturbations of membrane-related processes and redox-associated pathways could underlie the inhibitory effects on *F. proliferatum*. Membrane system integrity is critical for fungal survival, normal growth, and material transport, and serves as an important target for biological control agents [54]. In subsequent KEGG and qRT-PCR analysis, genes involved in the transport of substances including ions, amino acids, and pyridoxine were repressed by xd3 (Figure 8 and Figure 9). This finding raises the hypothesis that perturbations in membrane-associated processes and transmembrane transport could contribute to the inhibitory effects exerted by *B. velezensis* xd3 and its metabolites. This observation is highly consistent with previous reports by Huang et al. [55], Gao et al. [56], Wang et al. [57], and Wang et al. [58]. Nevertheless, further in vitro assays with purified compounds are required to clarify how individual metabolites affect fungal membrane homeostasis and gene expression.

Over-accumulation of reactive oxygen species (ROS) may induce irreversible injury to intracellular biomolecules (nucleic acids, proteins and lipids) within fungal cells, which constitutes a common antifungal mode-of-action for fungicides [59]. Pathogenic fungi have evolved sophisticated ROS-scavenging systems consisting of catalase, peroxidase, ascorbate peroxidase (APX), glutathione S-transferase (GST), superoxide dismutase (SOD), the tripeptide glutathione (GSH), and thioredoxin (TRX). These components function to alleviate cellular injury and sustain intracellular ROS homeostasis [60]. Previous studies have shown that biocontrol agents and their metabolites disrupt the redox homeostasis of fungi, leading to excessive accumulation of reactive oxygen species (ROS), which in turn affects their growth and pathogenicity [61,62]. In the present study, GO annotation revealed that genes associated with the oxidation–reduction process (GO:0055114) and oxidoreductase activity (GO:0016491) were significantly downregulated (Figure 7). KEGG enrichment analysis further indicated that pathways for metabolism of xenobiotics by cytochrome P450 (ko00980) and glutathione metabolism (ko00480) were also significantly repressed (Figure 8). Moreover, qRT-PCR data confirmed decreased expression of genes encoding thioredoxin (LB504_006262), endoplasmic reticulum oxidoreductase (LB504_004467), and mitochondrial intermembrane space protein (LB504_006530) (Figure 9), all of which are involved in fungal ROS homeostasis and pathogenicity [63,64,65]. Collectively, these transcriptional-derived findings raise the hypothesis that perturbations of redox-related pathways could constitute another potential contributor to the inhibitory effect of *B. velezensis* xd3 against *F. proliferatum*.

Meanwhile, GO annotation and KEGG enrichment analysis revealed that genes of *F. proliferatum* involved in RNA binding, ribosome biogenesis, ribonucleoprotein complexes, tRNA biosynthesis, transcriptional machinery, and transcription factors exhibited varying degrees of upregulation (Figure 7 and Figure 8). This phenomenon is consistent with a report by Zhong et al., which demonstrated that treatment with *B. velezensis* triggered the upregulation of genes associated with ribosome biogenesis and protein synthesis in *Colletotrichum scovillei* [66]. This phenomenon may be tentatively ascribed to multiple antagonistic metabolites secreted by strain xd3, which potentially target distinct intracellular sites in *F. proliferatum*. In response to cellular damage induced by these antimicrobial metabolites, *F. proliferatum* tends to enhance translational capacity via up-regulation of RNA synthesis and processing as well as pre-ribosome assembly, thereby generating larger amounts of functional proteins. This response represents a compensatory strategy adopted by *F. proliferatum* under xd3-induced stress. In short, *F. proliferatum* is relatively susceptible to metabolites produced by xd3. Transcriptomic analysis indicates that these metabolites may provoke transcriptional perturbations related to membrane integrity, membrane-transport activity, and cellular redox homeostasis in *F. proliferatum*. Nevertheless, further experiments are required to verify the occurrence of physiological membrane damage or redox imbalance.

## 5. Conclusions

In the present work, bacterial strain xd3 was isolated and identified as *B. velezensis* t based on 16S rRNA and gyrA gene sequencing. *B. velezensis* xd3 exhibits strong antagonistic activity against eight plant-pathogenic fungal species, especially *F. proliferatum*, the causal agent of garlic root rot. Treatment with *B. velezensis* xd3 markedly inhibited mycelial growth of *F. proliferatum* and triggered severe morphological alterations in fungal hyphae. Soil drench application of *B. velezensis* xd3 effectively alleviated garlic root rot symptoms caused by *F. proliferatum*. Biochemical assays confirmed that *B. velezensis* xd3 secretes cellulase, amylase, protease, and siderophores. Metabolomic profiling further revealed that *B. velezensis* xd3 may produce multiple antifungal metabolites, such as lipopeptides (putative surfactins b and c) and polyketides. Furthermore, *B. velezensis* xd3 induced substantial transcriptional changes in genes associated with membrane integrity, transmembrane transport and redox homeostasis in *F. proliferatum*. Collectively, *Bacillus velezensis* xd3 represents a promising biocontrol agent for the sustainable management of garlic root rot.

## Figures and Tables

**Figure 1:**
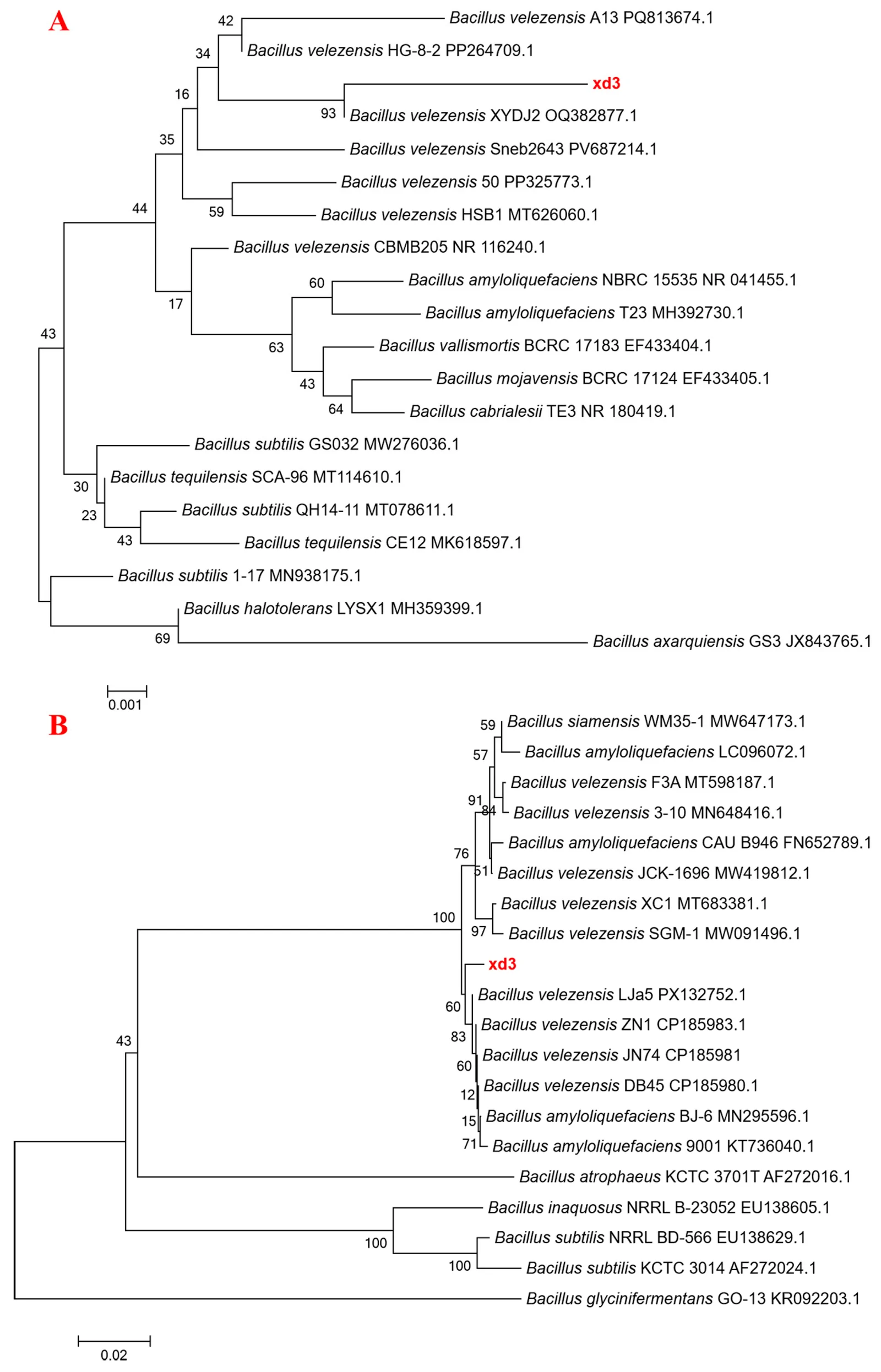
Molecular identification of strain xd3. (**A**) Phylogenetic tree of strain xd3 based on 16S rRNA sequences. (**B**) Phylogenetic tree of strain xd3 based on *gyrA* sequences.

**Figure 2:**
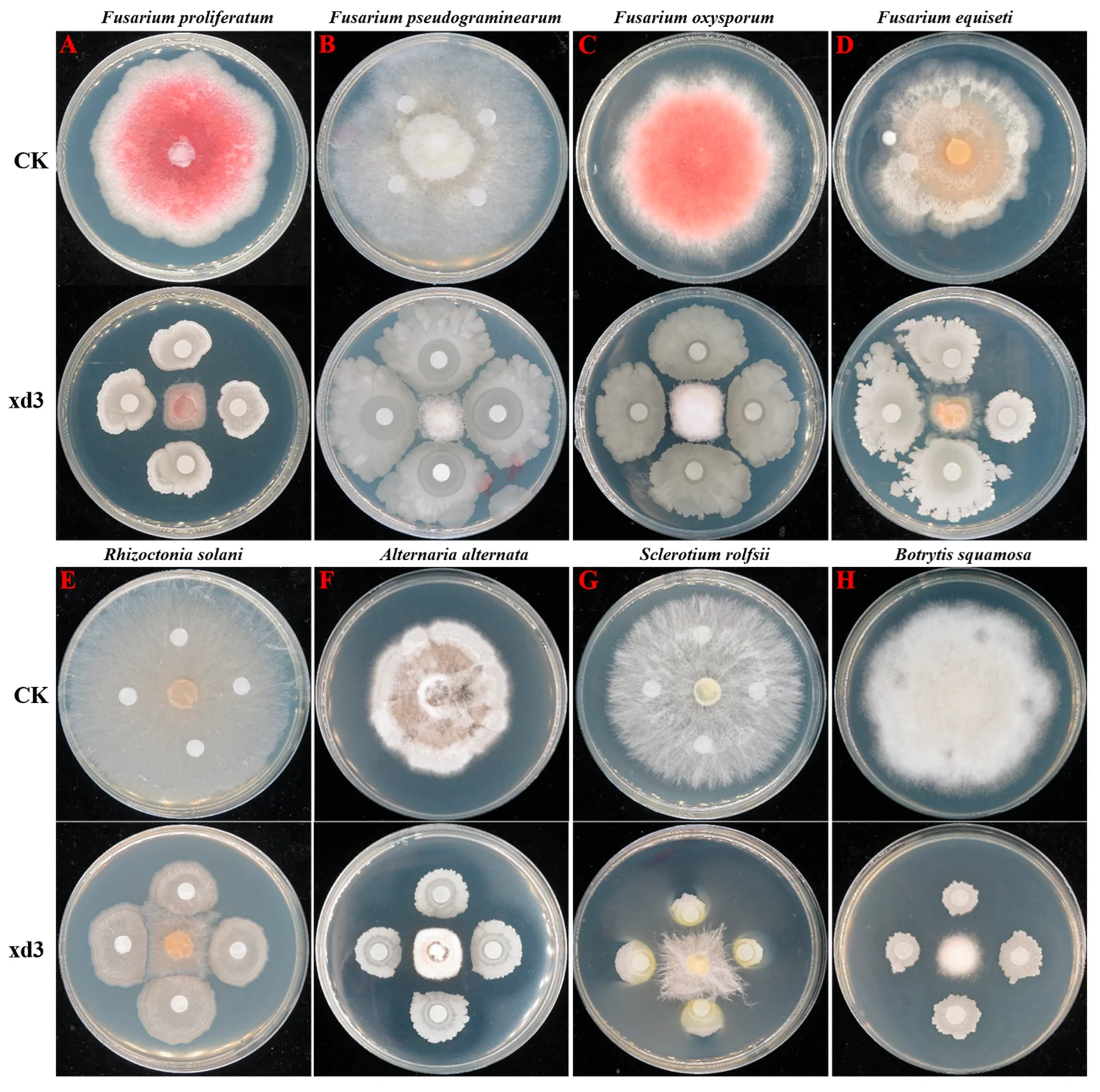
Inhibitory effects of *B. velezensis* xd3 against eight fungal phytopathogens. (**A**) *Fusarium proliferatum*; (**B**) *Fusarium pseudograminearum*; (**C**) *Fusarium oxysporum*; (**D**) *Fusarium equiseti*; (**E**) *Rhizoctonia solani*; (**F**) *Alternaria alternata*; (**G**) *Sclerotium rolfsii*; (**H**) *Botrytis squamosa*.

**Figure 3:**
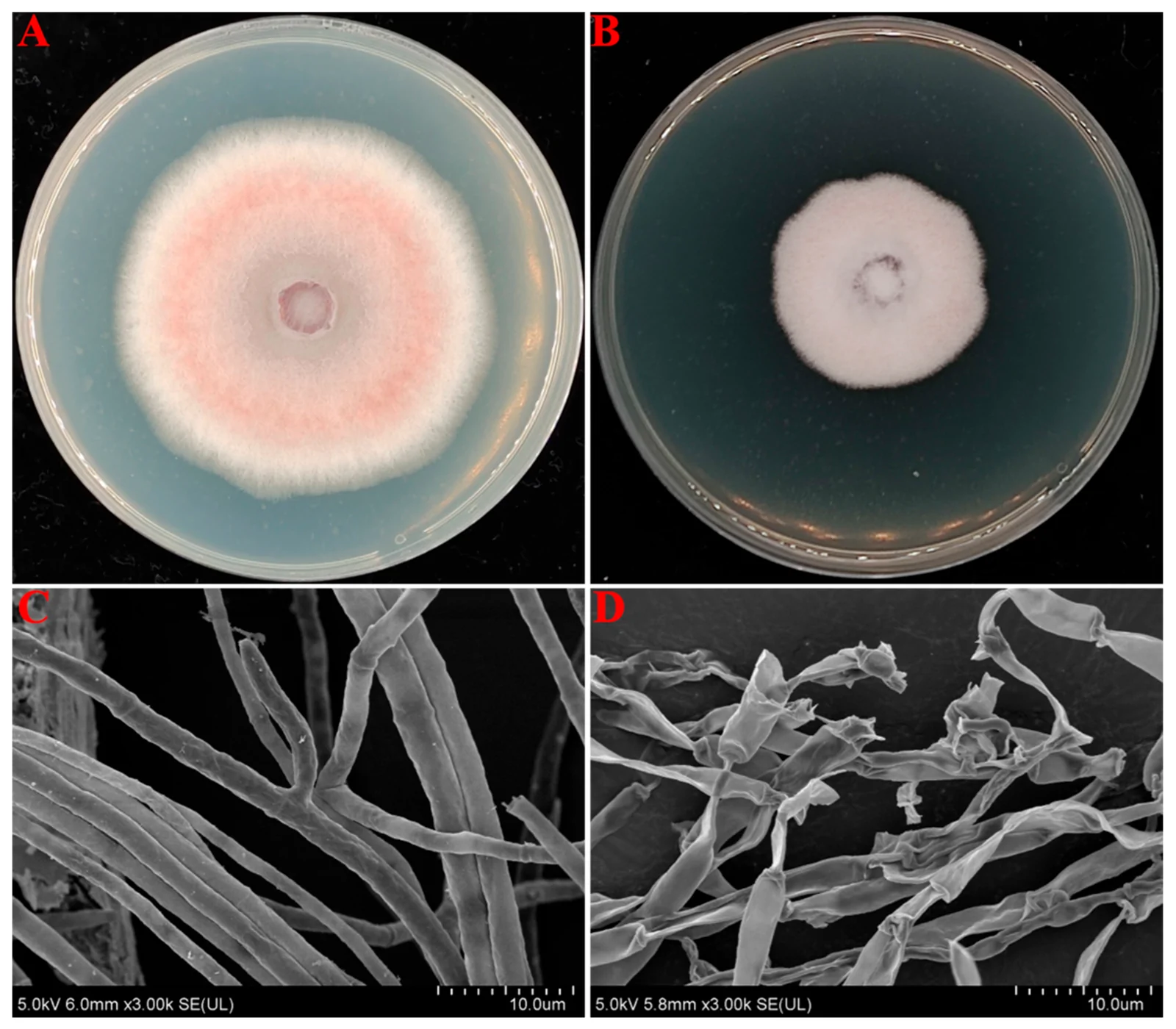
Inhibitory effect of biocontrol *B. velezensis* xd3 on *F. proliferatum*. (**A**,**B**) Growth status of *F. proliferatum* in the control group (**A**) and the cell-free fermentation broth-treated group (**B**). (**C**,**D**) Mycelial morphology of *F. proliferatum* in the control group (**C**) and the cell-free fermentation broth-treated group (**D**).

**Figure 4:**
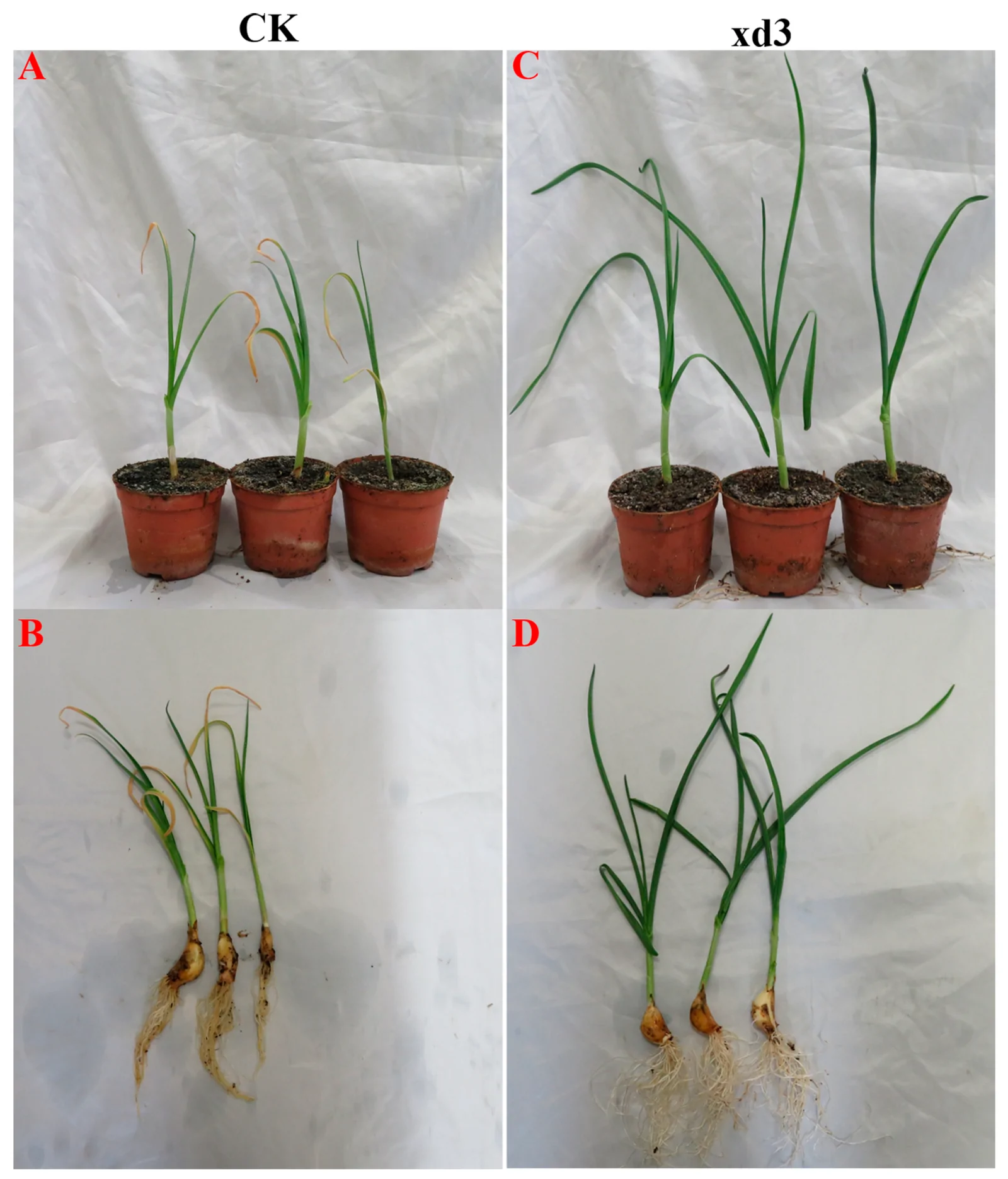
The treatment of *B. velezensis* xd3 significantly reduced garlic root rot symptoms. (**A**,**B**) Garlic root rot symptoms of control group. (**C**,**D**) Garlic root rot symptoms of xd3 treatment group.

**Figure 5:**
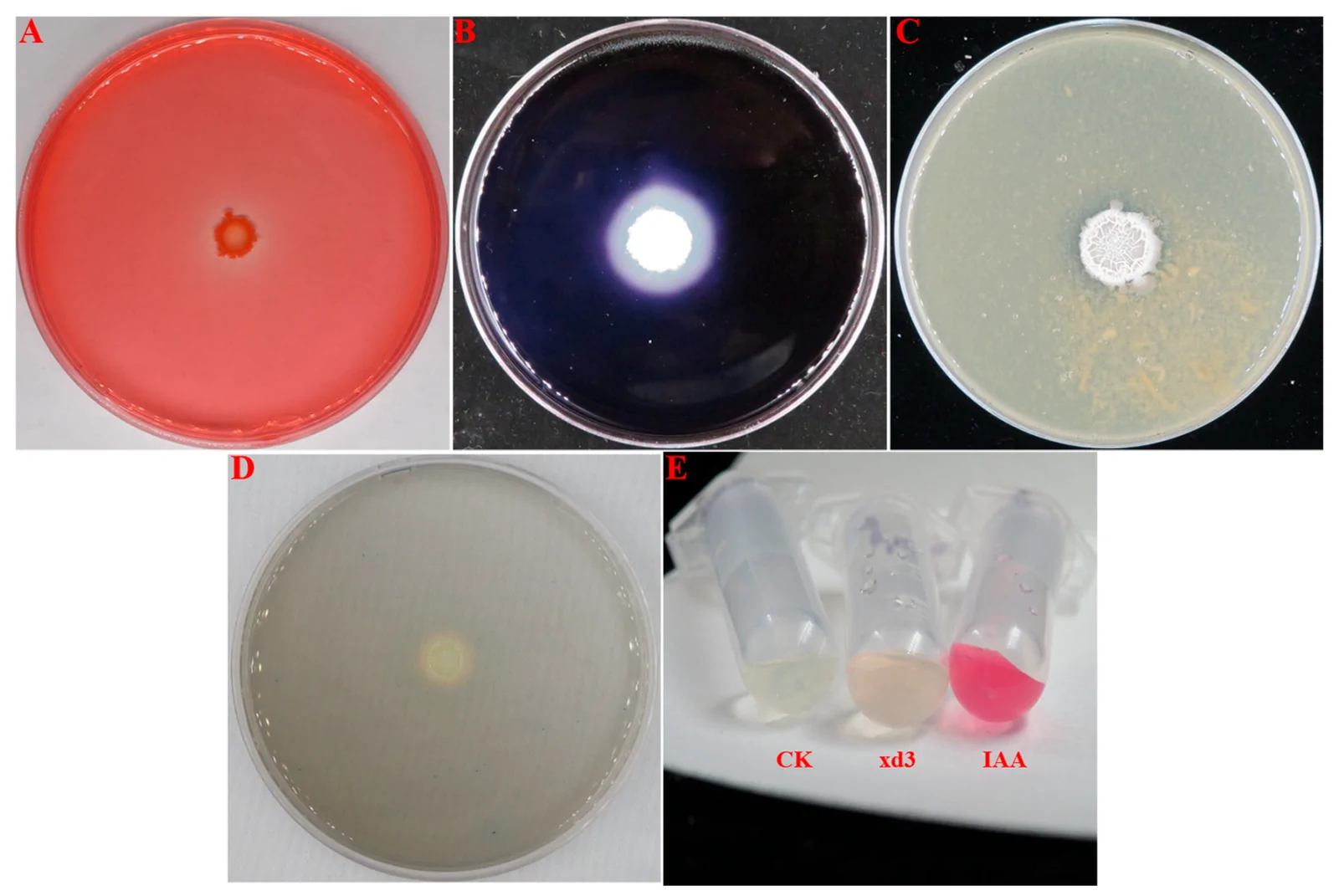
Biochemical characteristics of *B. velezensis* xd3. The isolate produced (**A**) cellulase, (**B**) amylase, (**C**) protease, (**D**) siderophores, and (**E**) IAA (from left to right: LB medium control, xd3, and IAA).

**Figure 6:**
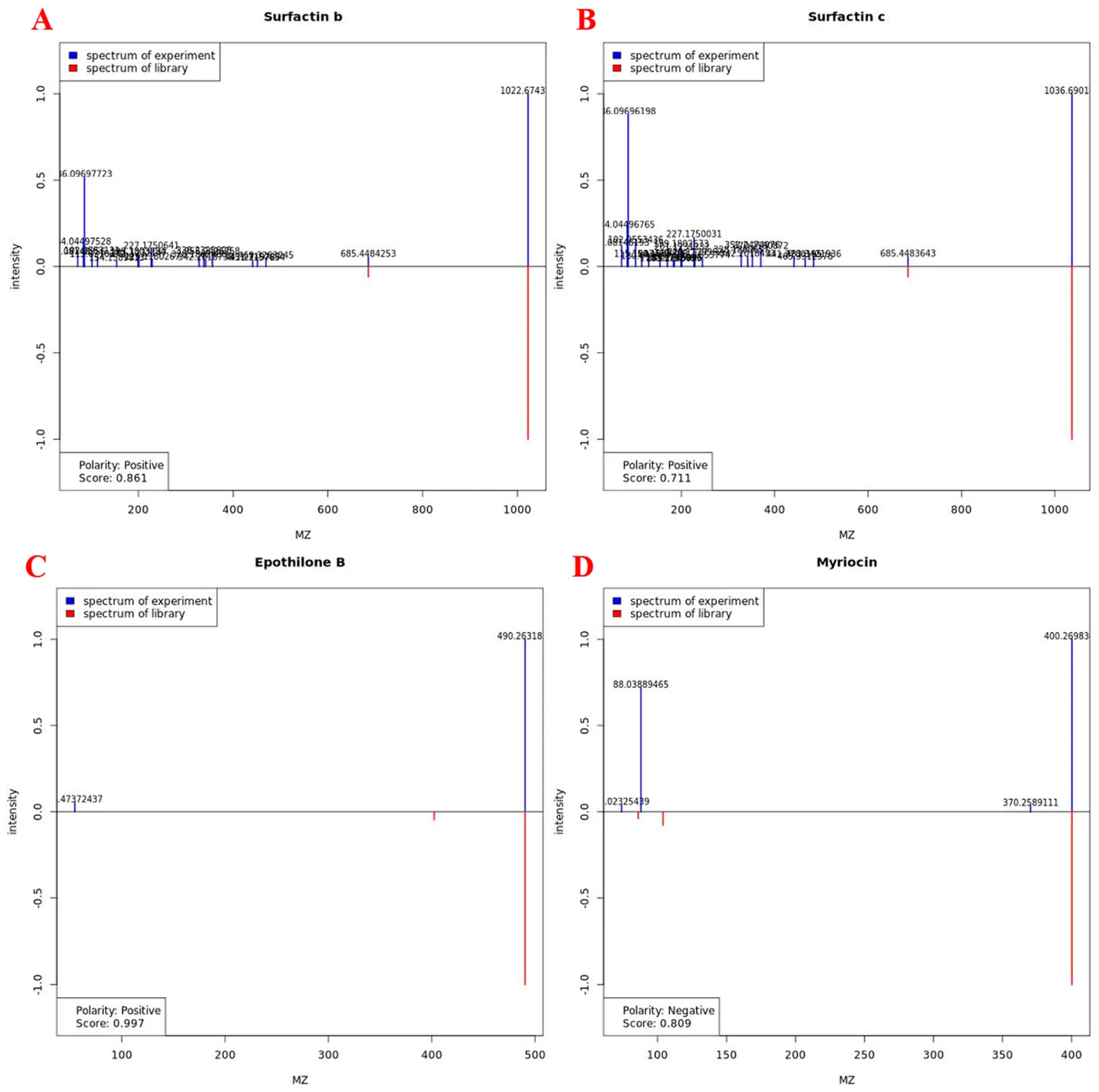
Putative annotation of bioactive metabolites from *B. velezensis* xd3 based on UHPLC-MS library matching. Representative mass-spectrum profiles for tentatively assigned surfactin b (**A**), surfactin c (**B**), epothilone B (**C**), and myriocin (**D**) are shown.

**Figure 7:**
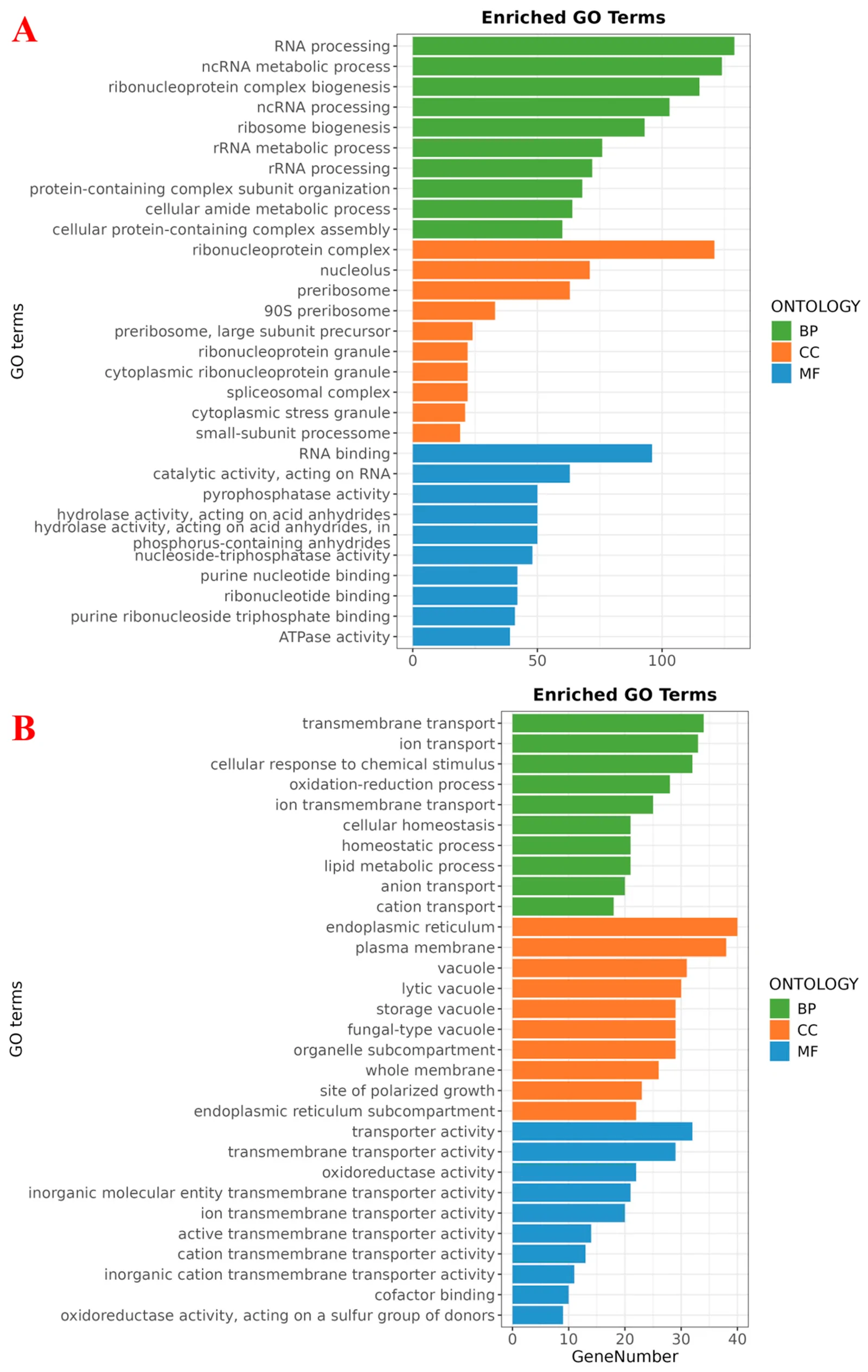
GO analysis of differential genes of the transcriptome regarding the effect of *B. velezensis* xd3 on *F. proliferatum*. (**A**) GO enrichment map of upregulated differentially expressed genes. (**B**) GO enrichment map of downregulated differentially expressed genes.

**Figure 8:**
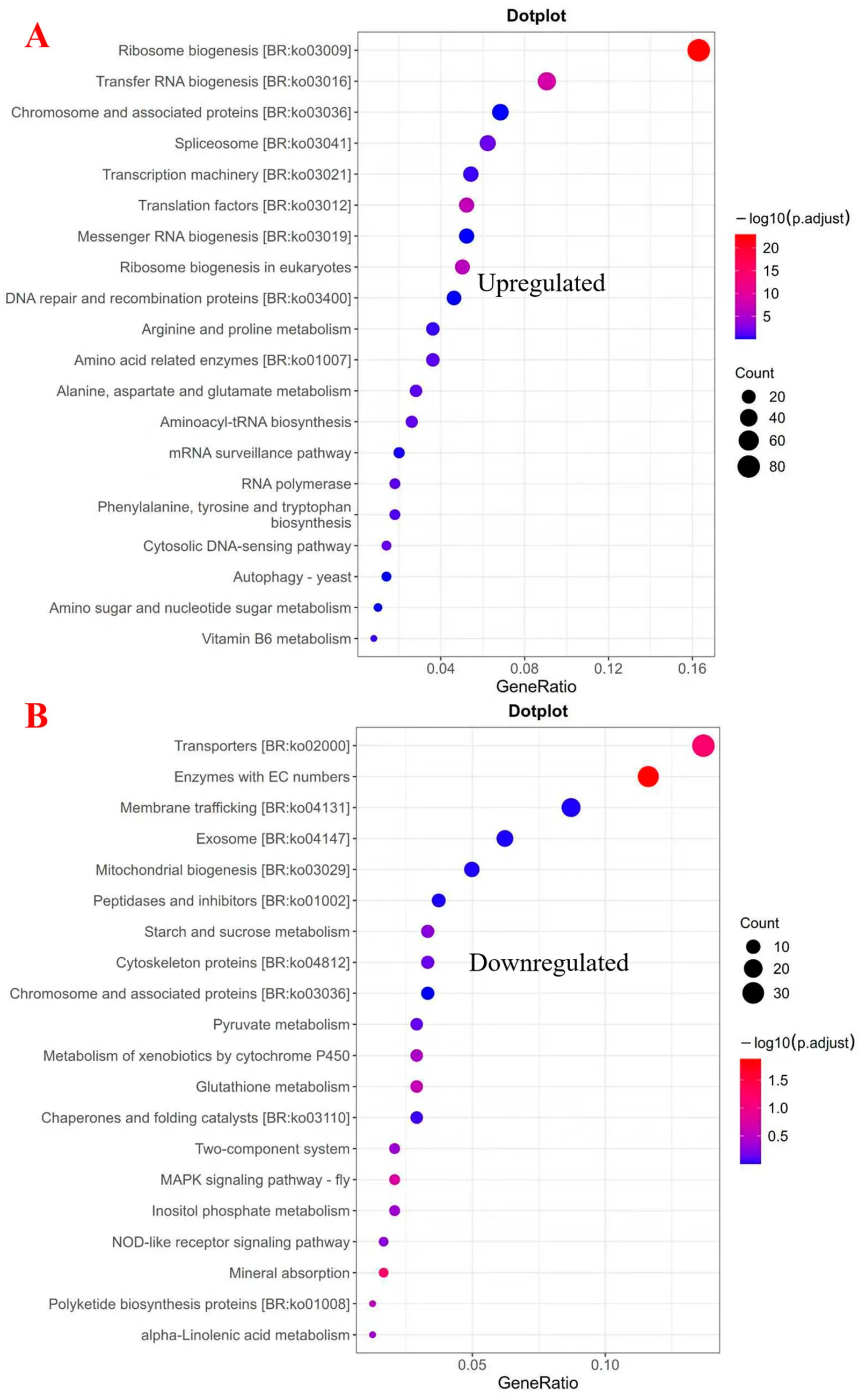
Bubble chart of KEGG enrichment analysis for differentially expressed genes. (**A**) KEGG enrichment pathways of upregulated differentially expressed genes; (**B**) KEGG enrichment pathways of down-regulated differentially expressed genes.

**Figure 9:**
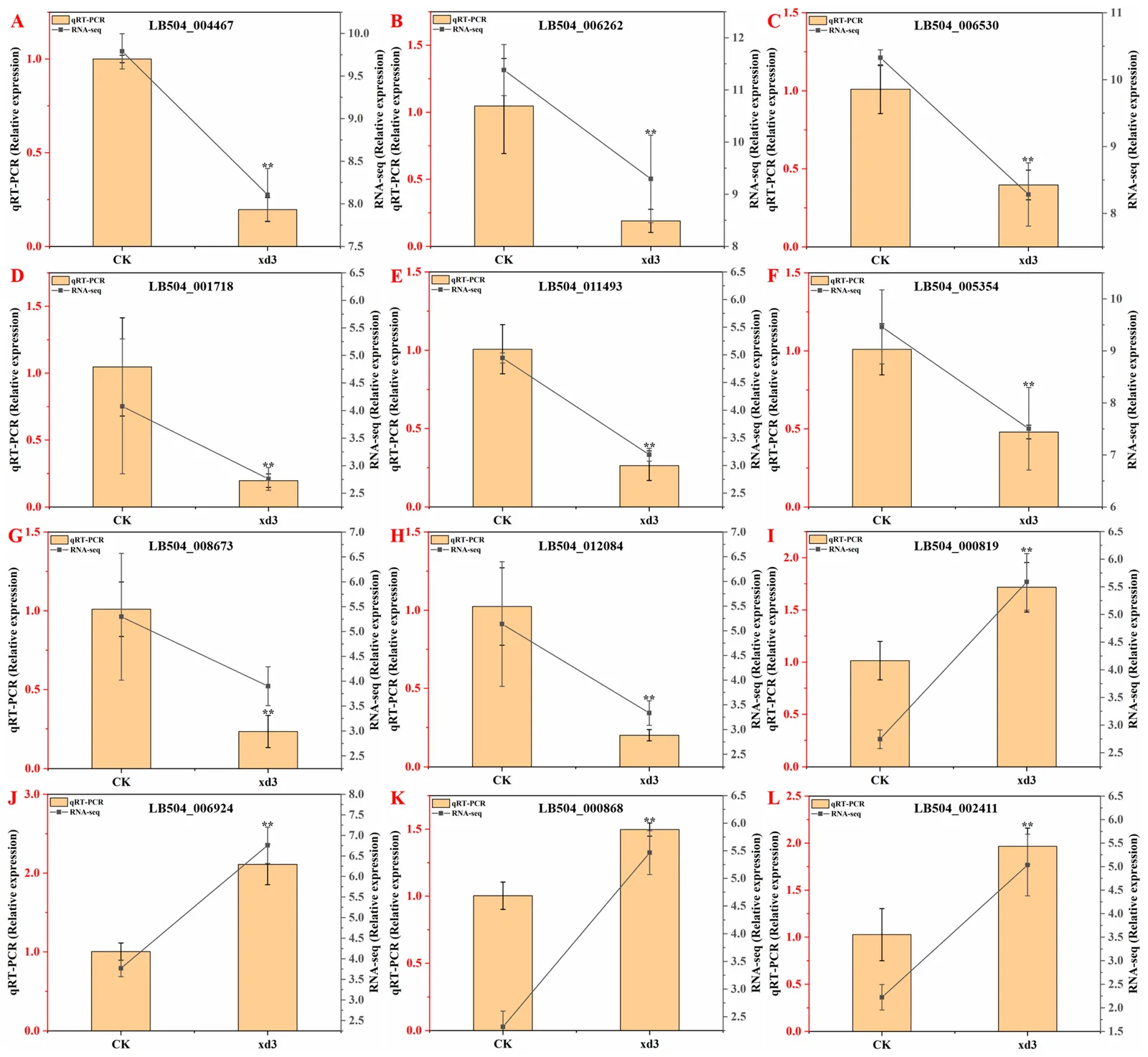
qRT-PCR analysis of selected DEG genes in *F. proliferatum* under *B. velezensis* xd3 treatment. (**A**) LB504_004467; (**B**) LB504_006262; (**C**) LB504_006530; (**D**) LB504_001718; (**E**) LB504_011493; (**F**) LB504_005354; (**G**) LB504_008673; (**H**) LB504_012084; (**I**) (LB504_000819; (**J**) LB504_006924; (**K**) LB504_000868; (**L**) LB504_002411. The bar chart shows the relative expression levels determined by qRT-PCR (left *y*-axis) and the line chart indicates the relative expression levels from RNA-Seq data (right *y*-axis). Asterisks denote significant differences (** *p* < 0.01).

**Table 1:** Inhibitory activities of *B. velezensis* xd3 against fungal pathogens.

Pathogen	Disease	Inhibition Rate (%)
*Fusarium proliferatum*	garlic root rot	74.80 ± 1.96 ab
*Fusarium pseudograminearum*	wheat crown rot	76.38 ± 3.53 ab
*Fusarium oxysporum*	cotton fusarium wilt	74.88 ± 2.59 ab
*Fusarium equiseti*	soybean root rot	78.57 ± 2.86 ab
*Rhizoctonia solani*	rice sheath blight	73.52 ± 1.36 ab
*Alternaria alternata*	tobacco brown spot	69.94 ± 0.49 ab
*Sclerotium rolfsii*	peanut southern blight	73.64 ± 2.05 ab
*Botrytis squamosa*	leek gray mold	77.25 ± 1.89 ab

The data are shown as the mean ± standard deviation of three replicates, and the different letters represent significant differences (*p* < 0.05).

**Table 2:** Control effect of *B. velezensis* xd3 on garlic root rot.

Group	Disease Index (%)	Rate Relative Control Efficacy (%)
CK	65.88 ± 4.95 a	_
Xd3	26.45 ± 4.91 b	59.85

The data are shown as the mean ± standard deviation of three replicates, and the different letters represent significant differences (*p* < 0.05).

**Table 3:** Putative metabolite annotation of the sterile fermentation broth from *B. velezensis* xd3 based on UHPLC-MS library matching.

Retention Time (s)	Mass–Charge Ratio (*m*/*z*)	Ion Binding Mode	Compound Type	Relative Peak Area
272.62	1022.67	[M+H]+	Surfactin b	70,554,471.68
270.47	1036.69	[M+H]+	Surfactin c	139,091,975.6
230.47	490.26	[M+H-H_2_O]+	epothilone B	86,558,980.2178235
287.00	400.27	[M-H]-	myriocin	53,337,067.8204639
417.33	654.28	[M+H]+	ferrioxamine	9,323,368.30984453
755.84	587.35	[M+H]+	desferrioxamine D2	14,425,570.2104062
757.33	601.34	[M+H]+	nocardamine	20,454,656.5546667

## Data Availability

Data is contained within the article and Supplementary Materials.

## Supplementary Materials

The following supporting information can be downloaded at: https://www.mdpi.com/article/10.3390/jof12100721/s1. Figure S1: Identification and physiological characteristics of strain xd3; Figure S2: Types and relative proportions of metabolites in the sterile fermentation broth of *Bacillus velezensis* xd3; Figure S3: Putative annotation of bioactive metabolites from *B. velezensis* xd3 based on UHPLC-MS library matching; Figure S4: Volcano plot of differentially expressed genes and clustering heatmap of differentially expressed genes; Figure S5: GO enrichment analysis plot of differentially expressed genes; Figure S6: KEGG enrichment analysis of differentially expressed genes; Table S1: Primers used in this study; Table S2: Sequences in this study; Table S3: Putative annotation of bioactive metabolites from *B. velezensis* xd3 based on UHPLC-MS library matching; Table S4: A list of genes and their encoded proteins. The raw RNA-seq data (PRJCA074583) reported in this paper have been deposited in the Genome Sequence Archive (Genomics, Proteomics & Bioinformatics 2021) in the National Genomics Data Center (Nucleic Acids Res 2022), China National Center for Bioinformation/Beijing Institute of Genomics, Chinese Academy of Sciences, and are publicly accessible at https://ngdc.cncb.ac.cn/gsa (accessed on 30 August 2026). In addition, raw RNA-seq data may be obtained via email from either the first author or the corresponding author.
