# Supplementary material for: Biocontrol Potential of Bacillus velezensis xd3 in Alleviating Root Rot of Garlic (Allium sativum L.)
Source: J Fungi (Basel). 2026 Sep 24;12(10):721. doi: 10.3390/jof12100721 (PMC13706299; doi:10.3390/jof12100721)
Supplement: Supplementary file 1 [file jof-12-00721-s001.zip › Supplementary Files.pdf]

*Supplementary Files*

# Biocontrol Potential of *Bacillus velezensis* xd3 in Alleviating Root Rot of Garlic (*Allium sativum* L.)


**Yutao Zhu [1,*], Xidie Liu [1], Meizi Han [1], Xianyang Wang [1], Jiangnan Zhou [1], Ganlin Tang [1], Tao Zhu [1], Mei Zhao [1], Taotao Li [1], Chunli Liao [1], Peifang Ma [2], Donghai Chen [3] and Lianzhe Wang [1,*]**

[1] Engineering Technology Research Center for Green Prevention and Control of Pests on Characteristic Crops in Pingdingshan, College of Life Science and Engineering, Henan University of Urban Construction,
Pingdingshan 467036, China; 17516557933@163.com (X.L.); 17638213933@163.com (M.H.); 13839726250@163.com (X.W.); 17516598687@163.com (J.Z.); 15753972901@163.com (G.T.); 30110201@hncj.edu.cn (T.Z.); meizhao2021@163.com (M.Z.); ltt198906@163.com (T.L.); liao20130427@163.com (C.L.)

[2] Pingdingshan Academy of Agricultural Sciences, Pingdingshan 467003, China; 15637530201@163.com

[3] Zhengzhou Academy of Agricultural and Forestry Science, Zhengzhou 450006, China; 362806722@163.com

* Correspondence: zyt17494@163.com (Y.Z.); jijy99@126.com (L.W.)


## 1 Supplementary Figures and Tables

### 1.1 Supplementary Figures

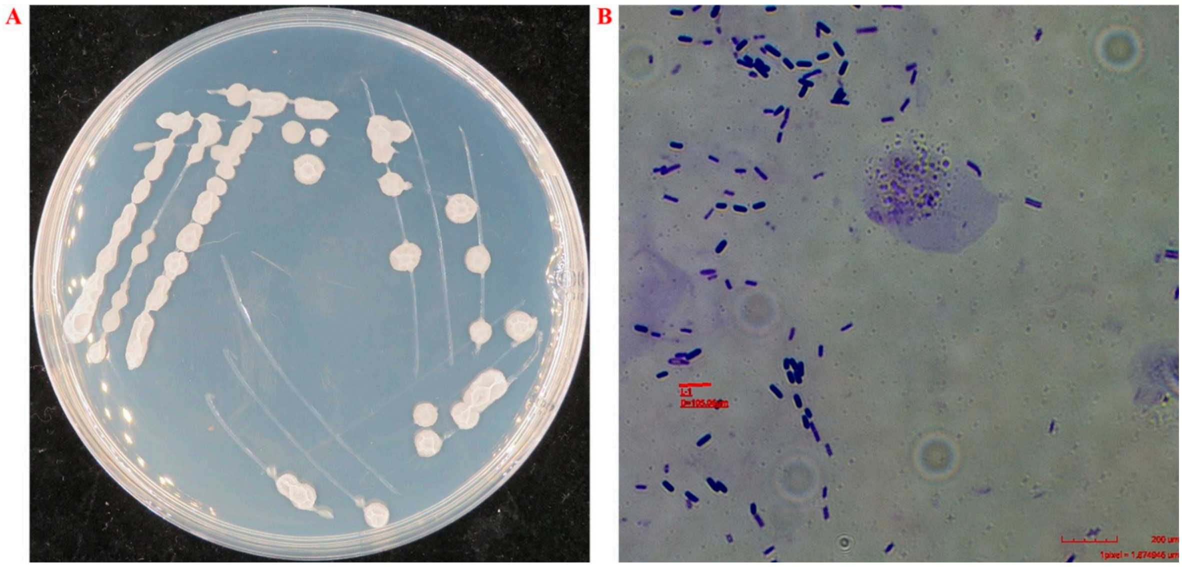


**Supplementary Figure S1.** Identification and physiological characteristics of strain xd3. **(A)** Observation of the apparent morphology of strain xd3 in LB medium. **(B)** Gram staining of strain xd3.

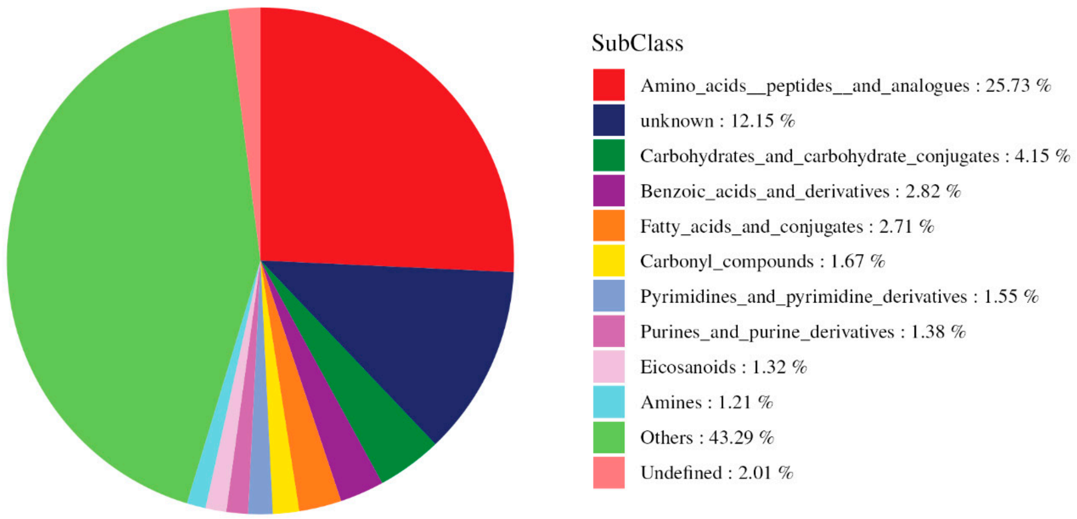


**Supplementary Figure S2.**

Types and relative proportions of metabolites in the sterile fermentation broth of *Bacillus velezensis* xd3.

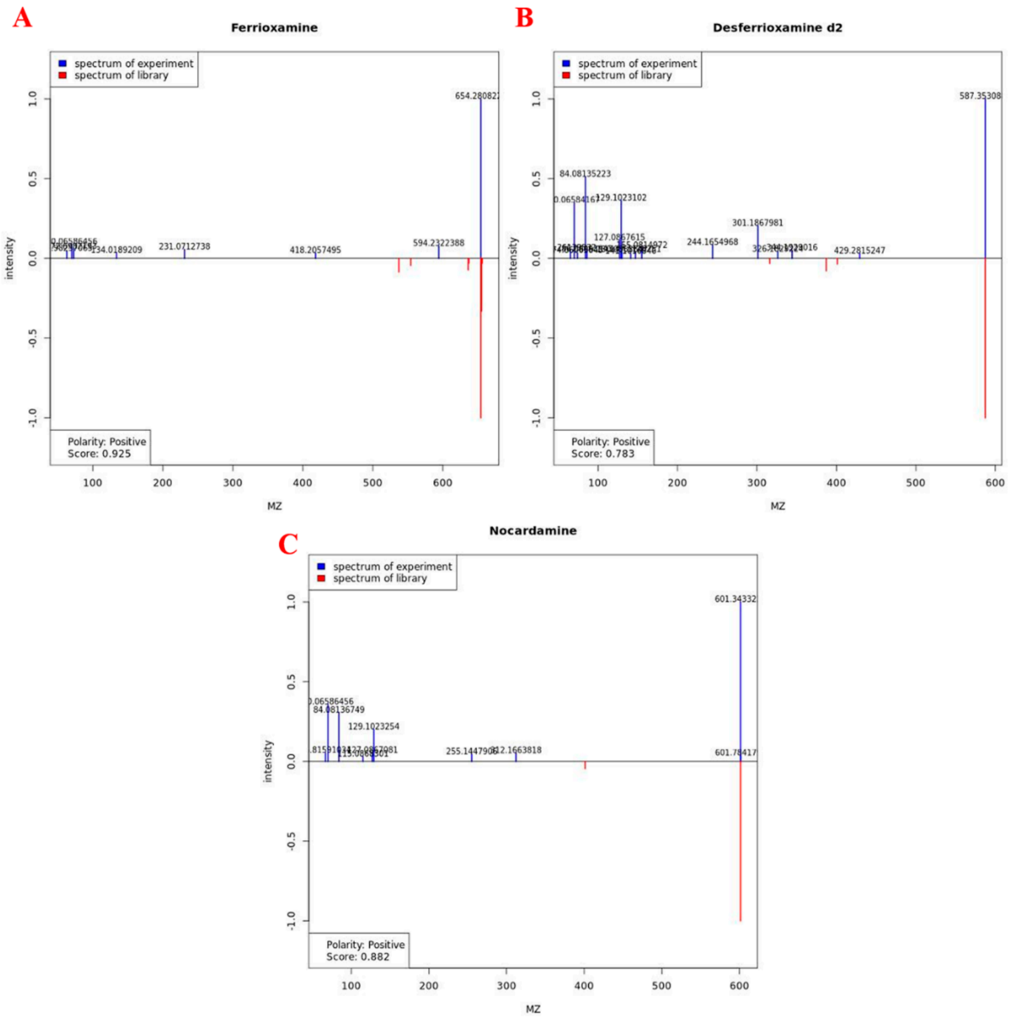


**Supplementary Figure S3.**

Putative annotation of bioactive metabolites from *B. velezensis* xd3 based on UHPLC-MS library matching. Representative mass-spectrum profiles for tentatively assigned ferrioxamine (A), desferrioxamine D2 (B), and nocardamine (C) are shown.

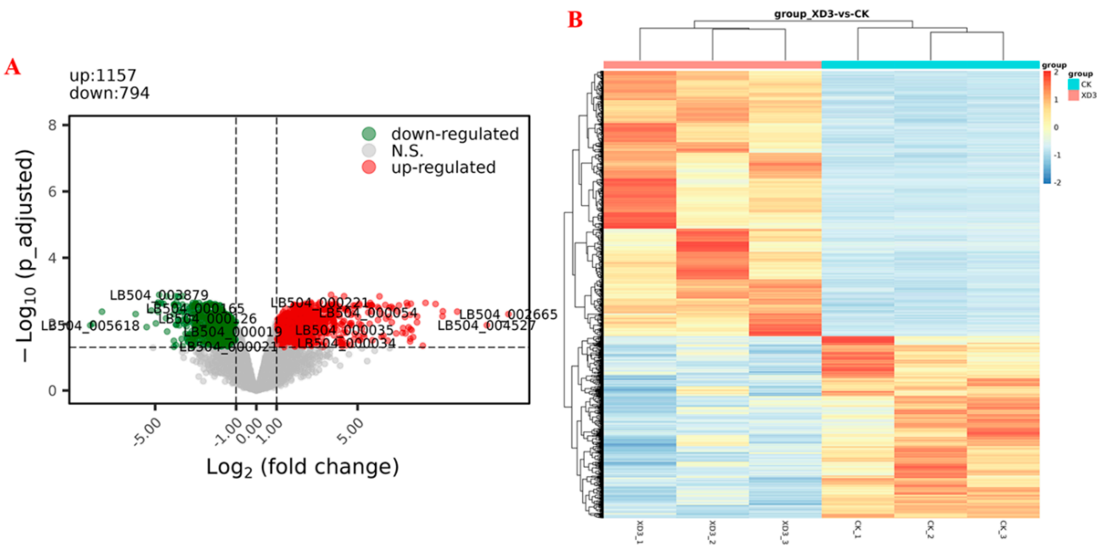


**Supplementary Figure S4.**

Volcano plot of differentially expressed genes (A) and clustering heatmap of differentially expressed genes (B).

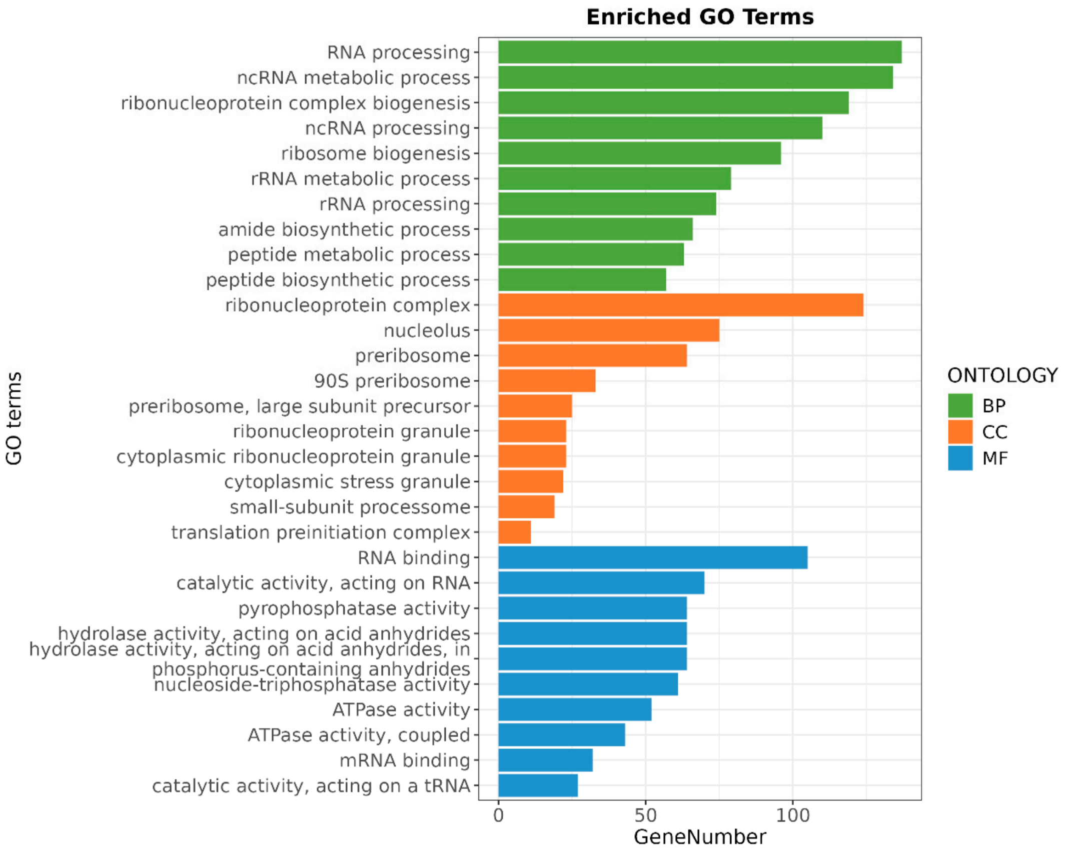


**Supplementary Figure S5.**

GO enrichment analysis plot of differentially expressed genes.

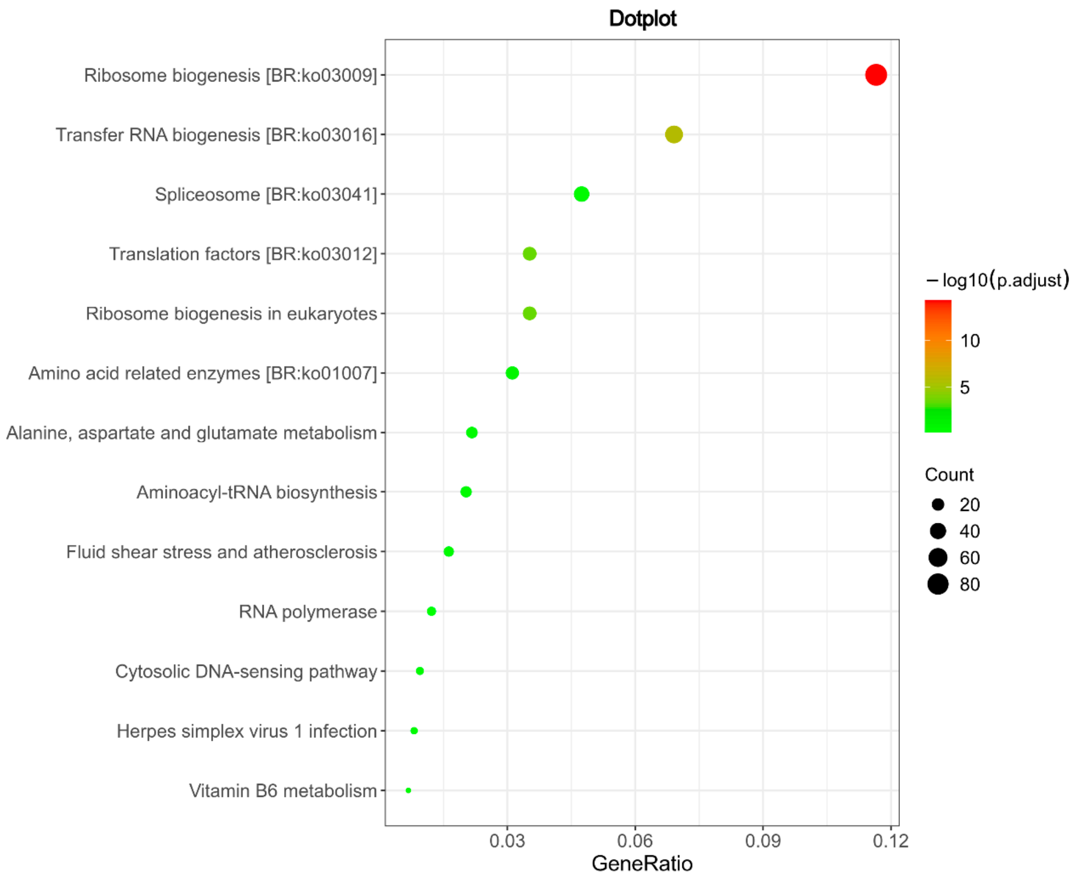


**Supplementary Figure S6.**

KEGG enrichment analysis of differentially expressed genes.
